# Recurrent Carotid Blowout Syndrome Managed by Infratemporal Internal Carotid Ligation: A Case Report

**DOI:** 10.1155/crot/2095726

**Published:** 2026-05-27

**Authors:** Hung-Chang Chen, Shih-An Liu, Man-Wei Hua

**Affiliations:** ^1^ Department of Medical Education, Taichung Veterans General Hospital, Taichung, Taiwan, vghtc.gov.tw; ^2^ Department of Otolaryngology, Taichung Veterans Hospital, Taichung, Taiwan; ^3^ Institute of Clinical Medicine, National Yang Ming Chiao Tung University, Taipei, Taiwan, nctu.edu.tw

**Keywords:** carotid blowout syndrome, case report, extracranial internal carotid artery, infratemporal approach, surgical ligation

## Abstract

Carotid blowout syndrome (CBS) is a rare but life‐threatening complication of head and neck cancer, with endovascular intervention as the preferred treatment, while surgical ligation remains critical when endovascular management is not feasible. We describe a 59‐year‐old man with oropharyngeal carcinoma (cT4bN0M0) who had achieved clinical remission after concurrent chemoradiation. Two months after completing treatment, he experienced his first episode of CBS. As immediate endovascular intervention was not feasible at that time, surgical ligation of the left common carotid artery (CCA) was performed as a life‐saving measure. He remained stable for 2 months until massive oral bleeding recurred, originating from a pseudoaneurysm of the extracranial internal carotid artery (ICA) distal to the previous ligation. As the proximal CCA had already been ligated and thus occluded, endovascular management was not possible in the second instance of CBS. Therefore, surgical ligation was performed via a preauricular infratemporal approach with intraoperative navigation. The pseudoaneurysm was successfully controlled by ligating the proximal and distal feeding vessels. The patient remained free from rebleeding for 3 years. This case demonstrates that infratemporal ICA ligation is a feasible and life‐saving option when CBS occurs distal to a previously sacrificed carotid artery and endovascular treatment is not practical.

## 1. Introduction

Carotid blowout syndrome (CBS), defined as the rupture of the carotid artery or its main branches, is a rare but life‐threatening complication in patients with head and neck cancer. CBS is clinically classified into three types: threatened (Type I), characterized by carotid exposure without bleeding; impending (Type II), presenting as sentinel hemorrhage; and acute (Type III), manifesting as massive arterial rupture. The pathophysiology is multifactorial and includes tumor invasion, radiation‐induced endothelial injury, progressive vessel wall necrosis, infection, and surgical trauma [1], all of which contribute to arterial wall fragility and pseudoaneurysm formation. Computed tomography angiography (CTA) can provide important diagnostic signs in patients with impending CBS and may help improve clinical outcomes through early detection and intervention [2].

The reported incidence of CBS ranges from approximately 3%–4.5% among head and neck cancer patients, with mortality rates exceeding 40%–50%, particularly in acute presentations [3]. Risk factors include hypopharyngeal and oropharyngeal primary tumors, prior to high‐dose radiotherapy, open cervical wounds, skull base involvement, and recurrent disease [4]. Common treatment options include surgical ligation, endovascular embolization, and reconstruction with stent grafts [3]. Among these, endovascular management is now the preferred approach due to its lower complication rates compared to surgical intervention. However, surgical ligation remains indispensable when endovascular access is not feasible. Management of CBS requires careful evaluation of collateral circulation, carotid occlusion testing, and perioperative airway management, as these factors may influence postoperative neurological outcomes [5, 6].

We present a case of recurrent CBS in a patient with oropharyngeal squamous cell carcinoma who had achieved clinical remission after concurrent chemoradiation. Following prior ligation of the common carotid artery (CCA), recurrent hemorrhage from the extracranial internal carotid artery (ICA) was successfully managed via a preauricular infratemporal surgical approach. This case is reported in accordance with the CARE guidelines [7].

## 2. Case Report

**FIGURE 1:**
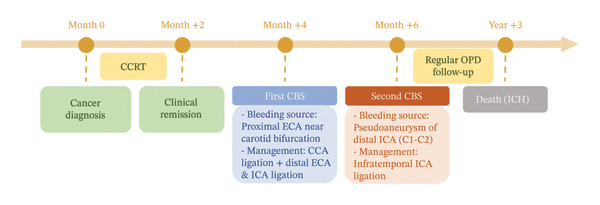
Clinical timeline of recurrent carotid blowout syndrome (CBS). Following cancer diagnosis and concurrent chemoradiotherapy (CCRT), the patient achieved clinical remission. Two months later, the first CBS episode occurred, with bleeding originating from the proximal external carotid artery (ECA) near the carotid bifurcation, managed by common carotid artery (CCA) ligation with distal ECA and internal carotid artery (ICA) ligation. A second CBS episode developed at 4 months due to a pseudoaneurysm of the distal ICA (C1‐C2 segment), which was treated with infratemporal ICA ligation. The patient subsequently underwent regular outpatient follow‐up and remained free from rebleeding for 3 years before death due to intracranial hemorrhage (ICH).

This 59‐year‐old man with p16‐negative oropharyngeal squamous cell carcinoma involving the nasopharynx and hypopharynx near the skull base (cT4bN0M0) received concurrent chemoradiation consisting of avelumab and IMRT to a total dose of 70 Gy in 35 fractions with cisplatin (100 mg/m^2^), achieving clinical remission. He had smoked two packs per day for over 20 years and quit after diagnosis, with a BMI of ∼19 kg/m^2^. A timeline summarizing the clinical course is presented in Figure 1.

### 2.1. First CBS Episode

**FIGURE 2:**
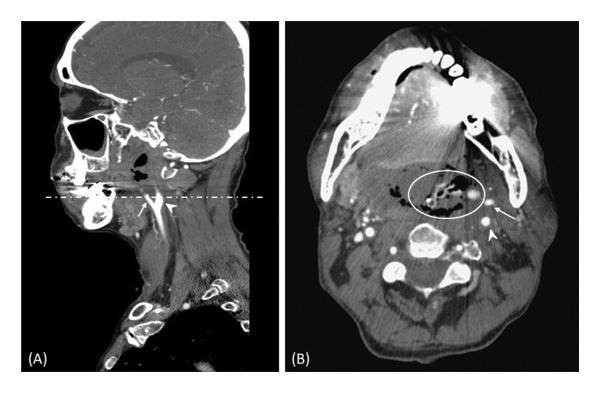
First episode of carotid blowout syndrome. Contrast‐enhanced computed tomography angiography (CTA) in (A) sagittal and (B) axial views reveals extensive necrotic tissue infiltrating the left carotid artery. The circle highlights active contrast extravasation originating from the proximal segment of the left external carotid artery (ECA). Arrows indicate the ECA, and arrowheads indicate the internal carotid artery (ICA). The dashed line in (A) denotes the corresponding axial level (B).

Two months after completing radiotherapy, the patient presented to the emergency department with massive bleeding from the nose, mouth, and tracheostomy tube. His hemoglobin level dropped from 13.6 to 8.3 g/dL. CTA disclosed the left ICA infiltrated by necrotic tissue with extravasation from the left external carotid artery (ECA) (Figure 2), consistent with acute CBS (Type III).

Due to the life‐threatening hemorrhage and the unavailability of immediate endovascular intervention, as the interventional radiologist was engaged in another emergent procedure, urgent surgical intervention was performed. The patient and family were informed of the high risk of ischemic stroke associated with carotid artery ligation. Through shared decision‐making, the patient elected to proceed with the operation as an emergent measure. The procedure was performed through a standard cervical approach under general anesthesia via the existing tracheostomy. Since the extravasation site was located at the very proximal segment of the ECA, and both the proximal ECA and ICA appeared heavily infiltrated by necrotic tissue, ligation of the CCA was necessary to achieve definitive hemostasis. Distal ligation of both the ECA and ICA was also performed to prevent retrograde hemorrhage. Preoperative balloon occlusion testing and cerebral perfusion evaluation were not done due to clinical urgency, as the results would not have altered the decision for immediate life‐saving hemostasis.

Hemodynamic stability was supported by aggressive fluid resuscitation. Intraoperative blood loss was minimal; the patient received 2 units of packed red blood cells (RBCs) intraoperatively, following 10 units administered preoperatively. Postoperatively, the patient was monitored in the ICU for 2 days and successfully weaned from mechanical ventilation within 24 h. Neurological examinations remained stable without focal deficits, with intact cranial nerve function and full motor strength in all extremities. He remained free from rebleeding for 2 months.

### 2.2. Recurrent CBS and Infratemporal Approach

**FIGURE 3:**
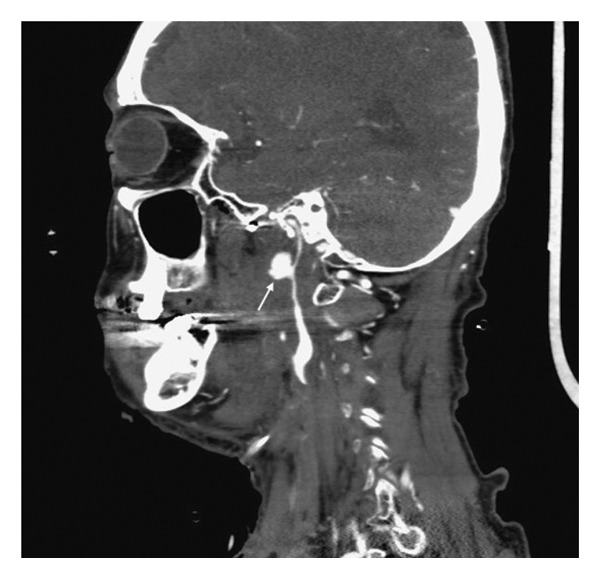
Second episode of carotid blowout syndrome. Computed tomography angiography (CTA) demonstrates a pseudoaneurysm of the left internal carotid artery (ICA) near the skull base (arrow), distal to the previously ligated common carotid artery (CCA).

2 months later, the patient experienced massive hemoptysis. Follow‐up CTA identified a pseudoaneurysm arising from the left ICA distal to the previous CCA ligation stalk, corresponding to the C1‐C2 segment of the ICA near the skull base (Figure 3). Acute CBS (Type III) was diagnosed, and an endovascular approach was unfeasible because prior CCA ligation precluded vascular access to the distal ICA lesion.

**FIGURE 4:**
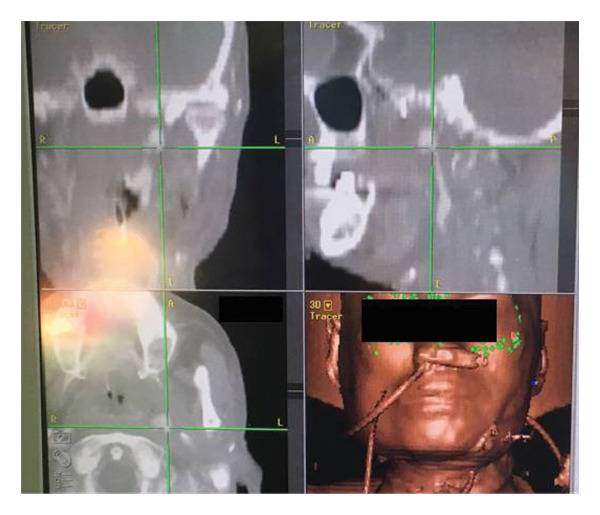
Intraoperative navigation system used to assist in surgical ligation of the pseudoaneurysm via an infratemporal approach during the second episode of carotid blowout syndrome. Multiplanar CT navigation images are shown in counterclockwise order from the upper right to the lower left (sagittal, coronal, and axial views), with three‐dimensional surface registration in the lower right panel. The green crosshairs in all planes are centered on the target pseudoaneurysm, demonstrating precise localization prior to ligation. (In the lower right panel, the rope‐like structures visible on the patient’s face represent the surgical navigation reference markers and registration cables, while the green dots indicate the tracked fiducial points used by the navigation system for spatial registration).

Surgical intervention was undertaken. Under the assistance of intraoperative navigation system, the standard parotidectomy incision was made over the left preauricular region (Figure 4). The facial nerve was preserved, and the deep lobe of the parotid gland was removed. The condylar head was resected using surgical saw, providing access to the skull base. The styloid process was identified with the carotid canal located medially. During dissection, the pseudoaneurysm ruptured unexpectedly. The proximal ICA stump and distal ICA segments near the carotid canal were immediately controlled with suture ligation. Intraoperative blood loss was approximately 580 mL, requiring 6 units of packed RBCs.

Following a 2‐day ICU stay, the patient was smoothly weaned from mechanical ventilation. Neurological examinations remained unremarkable, with preserved facial nerve function. A subsequent surgical site infection was successfully treated with a 3‐week course of intravenous piperacillin/tazobactam based on wound cultures (Klebsiella pneumoniae and Pseudomonas aeruginosa). The patient was discharged in stable condition, with outpatient follow‐up arranged every 2 weeks for 5 months and then monthly for 3 years.

### 2.3. Outcome

The patient remained free from bleeding for 3 years without recurrence. Ultimately, he experienced sudden death due to brainstem hemorrhage at another hospital. Detailed imaging information regarding the exact location and laterality of the hemorrhage was unavailable.

## 3. Discussion

Endovascular intervention is typically the first‐line treatment for CBS because of its high success rate, its association with lower rates of neurological complications is compared with open surgical ligation [8]. Embolization (59.3%) and stent placement (22.2%) are the most commonly employed techniques, while bypass grafting (8.3%) and surgical ligation (6.1%) are generally reserved for selected cases [9]. In addition, balloon test occlusion is often used in the preoperative evaluation to assess collateral cerebral circulation and tolerance to carotid artery sacrifice, thereby helping to predict the risk of ischemic complications following permanent vessel occlusion [10]. Despite the advantages of endovascular therapy, surgical ligation remains an essential therapeutic option and a last‐resort strategy when endovascular access is not feasible. Previous reports have demonstrated that surgical ICA ligation serves as a salvage strategy in complex CBS cases, especially in patients with prior vessel sacrifice, recently performed radical neck dissection, soft tissue necrosis, or active hemorrhage requiring urgent intervention [5]. Although commonly associated with a higher risk of neurological complications compared with endovascular therapy, surgical ligation can achieve definitive hemorrhage control with acceptable outcomes when acute bleeding [11]. These findings support the role of individualized treatment selection based on anatomical feasibility and clinical urgency.

In our case, the initial CBS episode presented with active, life‐threatening bleeding from friable, radiation‐damaged vessels with extensive necrosis. Because of the urgent clinical condition and the unavailability of immediate endovascular intervention—as the interventional radiologist was engaged in another emergent procedure—carotid artery ligation was undertaken as a life‐saving procedure. In addition, the active extravasation was located at the proximal ECA, in close proximity to the carotid bifurcation. Even if an endovascular approach had been attempted, embolization at this critical junction would have carried a substantial risk of unintended material migration or thromboembolism into the ICA, thereby increasing the risk of ischemic stroke. Furthermore, the extensive tissue necrosis and radiation‐induced vasculopathy not only rendered the vessel wall extremely fragile and unsuitable for stent‐graft placement but also likely contributed to progressive vascular injury and delayed pseudoaneurysm formation. In such a compromised vascular bed, endovascular interventions may carry a high risk of both technical failure and delayed complications, including stent infection or persistent rebleeding. Although open surgical ligation may not represent the optimal first‐line treatment compared to endovascular therapy, it provided definitive hemostasis in this case without immediate neurological sequelae.

Following this intervention, the development of a distal ICA pseudoaneurysm may be explained by a combination of radiation‐induced vasculopathy, ongoing tissue necrosis, and persistent hemodynamic stress from collateral circulation. Prior CCA ligation precluded both antegrade and retrograde endovascular approaches during the recurrent CBS episode, requiring distal ICA ligation via an infratemporal approach. This scenario underscores the value of skull base surgical exposure as a salvage strategy in selected patients with recurrent CBS when endovascular treatment is not feasible.

Surgical exposure of the ICA near the skull base is technically challenging due to its proximity to vital neurovascular structures. Historically, lesions above the Blaisdell line—a line drawn between the angle of the mandible and the tip of the mastoid process—were considered inaccessible using standard surgical techniques [12, 13]. Approaches to this region often result in disturbance to the facial nerve, temporal bone, and mandible, leading to substantial functional morbidity. In our case, the pseudoaneurysm was located at the distal cervical ICA near the petrous portion, corresponding to the C1‐C2 segments of the Bouthillier classification [14]. Adequate exposure in this area requires meticulous preoperative planning and often resection of surrounding bony structures such as the styloid, mastoid, and vaginal process [15]. These factors collectively underscore the technical demands and anatomical precision required for successful ICA exposure at the skull base.

Various techniques have been proposed to address these challenges, including transcervical, lateral infratemporal, midline mandibulotomy, and transnasal endoscopic approaches [12]. Among these, the lateral infratemporal approach offers favorable exposure with relatively low morbidity and excellent long‐term outcomes for ICA aneurysms [16]. In our case, we successfully performed ICA ligation using a preauricular lateral infratemporal approach with intraoperative navigation assistance, providing optimal visualization and precise control of the ICA while preserving facial nerve function and minimizing the risk to other cranial nerves. Our experience supports this technique as a reliable and safe last‐resort intervention for CBS when endovascular management is not an option.

Although the patient remained free from rebleeding for 3 years postoperatively, he eventually died from brainstem hemorrhage. The association between delayed intracranial hemorrhage and prior CBS management remains unclear, as most literature focuses on short‐term outcomes. A recent meta‐analysis reported a 29% rebleeding rate beyond 24 h following endovascular treatment, with higher recurrence in reconstructive procedures (30%) compared to deconstructive strategies (25%) [3]. To date, delayed intracranial hemorrhage or rebleeding events after 1 year following CBS in head and neck cancer patients are rarely reported. While a direct causal relationship between carotid ligation and subsequent brainstem hemorrhage cannot be proven, chronic hypoperfusion or collateral insufficiency may contribute. This case underscores the need for extended follow‐up and investigation into long‐term outcomes of both surgical and endovascular CBS management.

## 4. Conclusion

The case illustrates that when CBS occurs distal to a previously sacrificed carotid artery, surgical ligation via preauricular infratemporal approach can serve as a lifesaving intervention without major short‐term morbidity. It emphasized the role of surgical intervention as a last‐resort strategy when endovascular treatment is not feasible and highlights the importance of long‐term surveillance after CBS management.

## Funding

The authors have nothing to report.

## Ethics Statement

The study was conducted according to the Declaration of Helsinki. The study protocol was approved by the Institutional Review Board of the Taichung Veterans General Hospital (IRB no. CE25458B). Although the patient had passed away, informed consent for publication was obtained from the patient’s family. All clinical data and images have been fully anonymized to ensure protection of the patient’s privacy.

## Conflicts of Interest

The authors declare no conflicts of interest.

## Data Availability

The data that support the findings of this study are not publicly available to protect patient privacy but are available from the corresponding author upon reasonable request.
